# Uric acid mediates the relationship between mixed heavy metal exposure and renal function in older adult people

**DOI:** 10.3389/fpubh.2024.1403878

**Published:** 2024-07-22

**Authors:** Sai Pan, Yue Niu, Shuwei Duan, Delong Zhao, Qian Wang, Zheyi Dong, Guangyan Cai, Xiangmei Chen

**Affiliations:** Department of Nephrology, First Medical Center of Chinese PLA General Hospital, State Key Laboratory of Kidney Diseases, National Clinical Research Center for Kidney Diseases, Beijing Key Laboratory of Kidney Diseases Research, Beijing, China

**Keywords:** kidney function, uric acid, aging, heavy metals, machine learning

## Abstract

**Background:**

Population aging is a pivotal trend observed globally, and the exposure to heavy metals can exacerbate the aging process and lead to kidney damage. However, the impact of combined heavy metal exposure on renal function among older individuals remains elusive. Our study employs machine learning techniques to delve into the effects and underlying mechanisms of mixed exposure to heavy metals on the renal function of the aging population.

**Methods:**

This study extracted comprehensive data from the National Health and Nutrition Examination Survey (NHANES) conducted between 2015 and 2020. A total of 3,175 participants aged 60 years and above, with complete information on six metals – lead, cadmium, manganese, cobalt, mercury, and selenium, along with relevant covariates, were included in the study. To assess the impact of single or mixed metal exposure on the renal function of older adult individuals, various statistical techniques were employed: multiple logistic regression, weighted quantitative sum (WQS) regression, Bayesian kernel machine regression (BKMR), and mediation effects analysis.

**Results:**

Multiple logistic regression revealed that selenium and manganese were protective factors for chronic kidney disease (CKD). Cobalt was a risk factor for CKD. High concentrations of lead, cadmium, and cobalt were risk factors for urinary albumin creatinine ratio (ACR). WQS analyses revealed that mixed metal exposure was positively correlated with estimated glomerular filtration rate (eGFR) but negatively correlated with CKD. Selenium and manganese can neutralize the effects of other metals on eGFR. Mixed metal exposure was positively correlated with ACR, with lead and cadmium having a substantial effect. Mediation analysis showed that uric acid (UA) had a mediating effect of 9.7% and −19.7% in the association between mixed metals exposure and proteinuria and CKD, respectively.

**Conclusion:**

The impact of heavy metals on renal function in the older adult differs from that of adolescents and adults. This study suggests that elevated levels of mixed metals exposure are linked to proteinuria and CKD, with UA serving as a mediating factor.

## 1 Introduction

Population aging is a global concern. Globally, the population aged ≥60 years is estimated to be 9.01 million (12% of the total population); this number is expected to increase to 2 billion by 2050 because of the rise in life expectancy (1). By 2040, the number of Americans aged ≥65 years will reach 80.8 million, accounting for approximately 21.6% of the total population. Among them, the population aged ≥85 years will reach 14.4 million by 2040, an increase of 123% from 6.5 million in 2017 (2). In Europe, by 2060, the population aged ≥65 years will account for 28% of the European population (3). In China, by 2050, the population aged ≥65 years will increase to 400 million, accounting for 26.9% of the total population, of which the population aged ≥80 years will reach 150 million (4). During the aging process, the kidneys undergo progressive functional decline and macroscopic and microscopic histological changes, especially after 70 years of age (5). Despite significant structural and physiological changes, healthy older individuals seem to retain normal kidney function. As their renal reserve is substantially decreased, their kidneys are more susceptible to physiological, pathological, and toxicological challenges (6). However, there is relatively little research on the effect of environmental toxin exposure on the kidney health of older adult people.

Heavy metals are prevalent in various environmental media, such as air, soil, drinking water, and food; kidneys are important targets of heavy metal attacks (7). Older people may be exposed to toxic pollutants more frequently than in previous decades because of the extension of life expectancy and the increase in environmental pollution levels. They may also be exposed to higher levels of toxic pollutants, which increases the likelihood of kidney damage (8). Previous studies have found that metals and their combinations, including arsenic (As), lead (Pb), mercury (Hg), and cadmium (Cd), may affect kidney function in adolescents (9). Another study found that plasma levels of manganese (Mn), iron (Fe), and zinc (Zn) can prevent chronic kidney disease (CKD) in older people aged ≥90 years in long-lived areas (10). In individuals over 60 years of age, urinary copper (Cu) concentration is strongly positively correlated with ACR (11). The effect of heavy metals on renal function in older people might be significantly different from that in adults. However, research focusing on the effects of heavy metals on the renal function of older adult people is scarce. Furthermore, the role of metals in diseases often depends on their cooperation and interaction. A single metal is insufficient to comprehensively elucidate the occurrence and development of diseases. Therefore, it is worth exploring the effect of mixed metal exposure on the renal function of older adult people. In this study, we investigated the effects of six metals (Pb, Cd, Mn, cobalt (Co), Hg, and selenium (Se)) on renal function due to their known potential impacts on human health. These metals are commonly found in the environment and can be introduced into the body through various pathways, such as ingestion, inhalation, or skin contact (12). Exposure to these metals has been associated with a range of adverse health effects, including kidney damage, neurological problems, and developmental issues (13, 14). The potential pathophysiological mechanisms between metal exposure and renal health can be complex and multifaceted. Here are some possible pathways: oxidative stress, inflammation, direct toxicity, enzyme inhibition (15–17).

Moreover, the accumulation of uric acid (UA) in the body can lead to the occurrence of hyperuricemia or CKD (18). Moreover, there is a positive correlation between blood metal mixture and gout related results (19). However, there have been no studies reporting whether the effects of metal mixed exposure on the kidneys are mediated by UA. The aim of this study is to use cross-sectional survey data to elucidate the association between metal mixed exposure and kidney injury in the older adult, as well as the mediating role of UA.

## 2 Materials and methods

### 2.1 Study population

**Figure 1:**
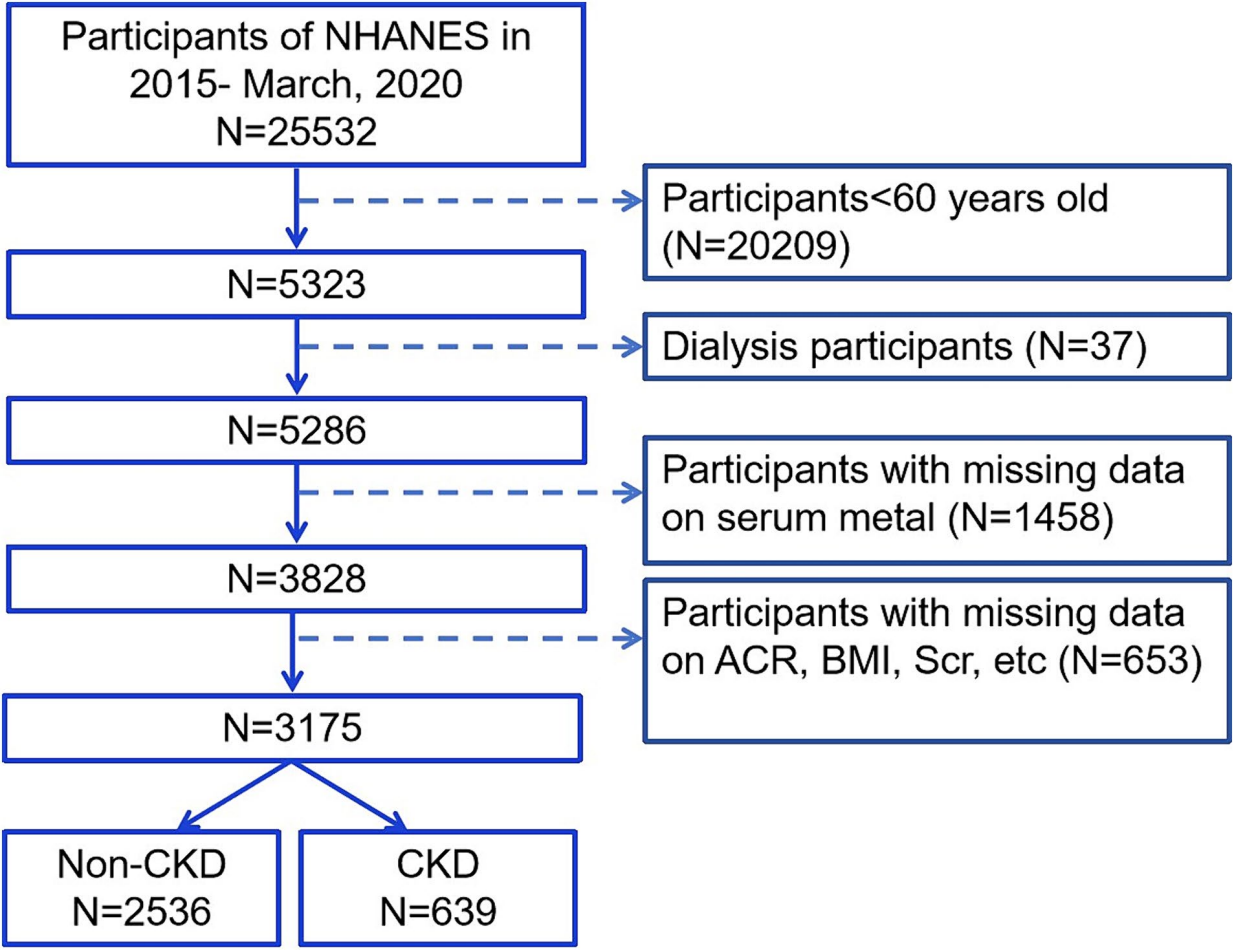
Flow chart of the inclusion subjects. Scr, serum creatinine; ACR, urinary albumin creatinine ratio; BMI, body mass index; CKD, chronic kidney disease.

The National Health and Nutrition Examination Survey (NHANES) is an ongoing nationwide cross-sectional survey with data available at the Centers for Disease Control and Prevention in the United States. This research protocol was approved by the Research Ethics Review Committee of the National Center for Health Statistics in the United States. All participants provided written consent during recruitment. We included data from older adult participants aged ≥60 years between March 2015 and March 2020 (*N* = 5,323). Next, we excluded dialysis participants (*N* = 37) and individuals lacking information on all six metals (*N* = 1,458). We also excluded individuals with missing data on serum creatinine (Scr), urinary albumin creatinine ratio (ACR), age, body mass index (BMI), alcohol consumption, and UA (*N* = 653). Finally, we enrolled 3,175 patients, of whom 639 were diagnosed with CKD (Figure 1).

### 2.2 Renal function assessment

We used estimated glomerular filtration rate (eGFR) and ACR to evaluate renal function. eGFR is based on the Chronic Kidney Disease Epidemiological Collaboration equation (20). The CKD-EPI equation, expressed as a single equation, is GFR = 141 × min(Scr/κ,1) ɑ × max(Scr/κ, 1)−1.209 × 0.993Age × 1.018 [if female] × 1.159 [if black], where Scr is serum creatinine, κ is 0.7 for females and 0.9 for males, ɑ is −0.329 for females and −0.411 for males, min indicates the minimum of Scr/κ or 1, and max indicates the maximum of Scr/κ or 1. We conducted continuous and binary result analyses on eGFR and ACR. As a binary result, eGFR below 60 mL/min/1.73 m^2^ was used to define CKD, and proteinuria was defined based on ACR ≥ 30 mg/g (21).

### 2.3 Measurement of blood chromium (Cr), cobalt (co), Pb, cd, Mn, selenium (se), and hg

All measurements of metal exposure were based on whole blood samples. All measurements were conducted at the National Center for Environmental Health and Centers for Disease Control and Prevention in Atlanta, Georgia. Cr and Co concentrations were measured using inductively coupled plasma mass spectrometry. However, Cr was not used in the analysis as Cr levels were below the minimum detection limit in 81.5% of the participants. Moreover, after the dilution sample preparation step, mass spectrometry was used to measure the concentrations of Pb, Cd, total Hg, Mn, and Se in whole blood. The detection rate and LLOD of the seven metals were presented in Table 1.

**Table 1:** The detection rate and LLOD of the seven metals.

Analyte description	LLOD	The detection rate
Cd, blood	0.10 μg/L	97.2%
Pb, blood	0.07 ug/dL	100.0%
Mn, blood	0.99 μg/L	100.0%
Hg, total, blood	0.28 ug/dL	84.1%
Se, blood	24.48 ug/dL	100.0%
Cr, blood	0.41 μg/L	18.5%
Co, blood	0.06 μg/L	96.7%

### 2.4 Statistical analysis

#### 2.4.1 Baseline statistical methods

All analyses were conducted using R (version 4.3.1). The demographic characteristics were compared between CKD status groups through chi-square test, *t*-test, and Mann–Whitney U test for categorical variables, normal continuous variables, and nonnormal continuous variables. Metal concentrations were categorized into four quartiles (Q1, Q2, Q3, and Q4) as categorical variables. Multivariate logistic regression was used to estimate the odds ratio (OR) and corresponding 95% confidence interval (CI) of metals related to CKD risk and ACR. All analyses were adjusted for age, gender, race/ethnicity, education, drinking status, BMI, smoking status, hypertension, and diabetes history.

#### 2.4.2 WQS

Weighted quantile sum (WQS) regression was used to explore the overall effect of metals on CKD and ACR, as it performs well in characterizing environmental mixtures. The R package gWQS ver 3.0.5 was used to calculate the WQS index, which comprises the weighted sum of individual metal concentrations based on experience. The WQS index (ranging from 0 to 1) represents the mixed exposure level of metals, and the components of interest are determined by non-negligible weights. The final result was explained as the synchronous effect of adding one quartile to the mixed metal on CKD and ACR.

#### 2.4.3 BKMR

Considering the potential nonlinear and nonadditive relationships among metals, Bayesian kernel machine regression (BKMR) was used to evaluate the mixed effects of all metals and the dose–response relationship between a single metal and CKD and ACR risks when fixing other metal concentrations (22). We mainly used the BKMR model to explore and visualize the following three exposure-response functions. (1) The univariate exposure-response function of single heavy metal exposure on CKD and ACR risk while fixing the other five metals at their corresponding median concentrations; (2) cumulative effects of metal mixtures at different quantiles on CKD and ACR risk compared to setting all mixtures at median concentrations; and (3) the posterior inclusion probability (PIP) of each metal in the mixture to determine the metal with the most significant effect on CKD risk.

We implemented a BKMR variable selection model with 10,000 iterations using the Markov Chain Monte Carlo algorithm. We then studied the convergence of the model by visually examining the trajectory map. The BKMR model was analyzed using the R package bkmr ver 0.2.2.

#### 2.4.4 Mediation analysis

Finally, mediation analysis allows us to calculate how many mediated effects are produced by other factors. In addition to providing statistical evidence for mechanistic analyses, this strategy is ideal for revealing pathways. The direct effect indicates the association between mixed metals and eGFR or ACR. The indirect effect indicates the association is mediated by other factors. The mediation ratio indicates the percentage of mediated effects. The WQS is a method for evaluating a mixture-outcome association by calculating a summary score for the mixture. If we consider the mixed metal exposure as a score, it becomes straightforward to treat it as any other variable. There are studies adopting similar methodologies, such as Wu et al. investigating the mediator between volatile organic compound co-exposure and kidney stones (23). Mediation analysis was performed using the R package mediation ver 4.5.0.

## 3 Results

### 3.1 Demographic characteristics

Of 3,175 older adult people, 639 were diagnosed with CKD (Table 2). The demographic characteristics of study participants with or without CKD. There were significant differences in age, race, BMI, education level, ACR, uric acid, hypertension, and diabetes history between the CKD and non-CKD groups.

**Table 2:** Characteristics of the study population.

	Overall	Non-CKD	CKD	*p*
**Number**	3,175	2,536	639	
**Albumin creatinine ratio (mg/g)**	10.20 (6.11, 22.38)	9.36 (5.88, 19.06)	14.89 (7.72, 54.84)	<0.001
**Gender (female,%)**	1,508 (47.5)	1,199 (47.3)	309 (48.4)	0.658
**Age (year)**	69.47 (6.82)	68.42 (6.48)	73.63 (6.54)	<0.001
**Race (%)**				<0.001
Mexican American	333 (10.5)	295 (11.6)	38 (5.9)	
Other Hispanic	363 (11.4)	322 (12.7)	41 (6.4)	
Non-Hispanic White	1,387 (43.7)	1,017 (40.1)	370 (57.9)	
Non-Hispanic Black	747 (23.5)	612 (24.1)	135 (21.1)	
Non-Hispanic Asian	244 (7.7)	210 (8.3)	34 (5.3)	
Other Race – including multi-racial	101 (3.2)	80 (3.2)	21 (3.3)	
**Education (%)**				0.002
Less than 9th grade	357 (11.2)	299 (11.8)	58 (9.1)	
9–11th grade (Includes 12th grade with no diploma)	360 (11.3)	288 (11.4)	72 (11.3)	
High school graduate/GED or equivalent	791 (24.9)	594 (23.4)	197 (30.8)	
Some college or AA degree	933 (29.4)	747 (29.5)	186 (29.1)	
College graduate or above	734 (23.1)	608 (24.0)	126 (19.7)	
**Body mass index (kg/m**^2^)	29.71 (6.45)	29.52 (6.43)	30.45 (6.48)	0.001
**Uric acid (umol/L)**	337.52 (86.59)	324.08 (79.84)	390.85 (91.69)	<0.001
**Alcohol user (%)**	2,680 (84.4)	2,143 (84.5)	537 (84.0)	0.819
**Smoker (%)**	1,560 (49.1)	1,234 (48.7)	326 (51.0)	0.307
**Hypertension (%)**	1,188 (37.4)	923 (36.4)	265 (41.5)	0.02
**Diabetes (%)**	819 (25.8)	605 (23.9)	214 (33.5)	<0.001
**Lead (ug/L)**	1.23 (0.84, 1.85)	1.20 (0.82, 1.81)	1.32 (0.93, 1.99)	<0.001
**Cadmium (ug/L)**	0.34 (0.22, 0.55)	0.33 (0.21, 0.54)	0.37 (0.24, 0.58)	0.003
**Mercury (ug/L)**	0.74 (0.38, 1.54)	0.76 (0.39, 1.58)	0.64 (0.34, 1.32)	<0.001
**Selenium (ug/L)**	185.13 (169.55, 202.37)	185.96 (170.70, 202.96)	181.34 (165.66, 199.96)	<0.001
**Manganese (ug/L)**	8.72 (7.04, 10.75)	8.82 (7.09, 10.88)	8.42 (6.78, 10.34)	0.001
**Cobalt (ug/L)**	0.14 (0.11, 0.18)	0.14 (0.11, 0.17)	0.16 (0.12, 0.22)	<0.001

### 3.2 Distribution and correlation of metals in the blood

**Figure 2:**
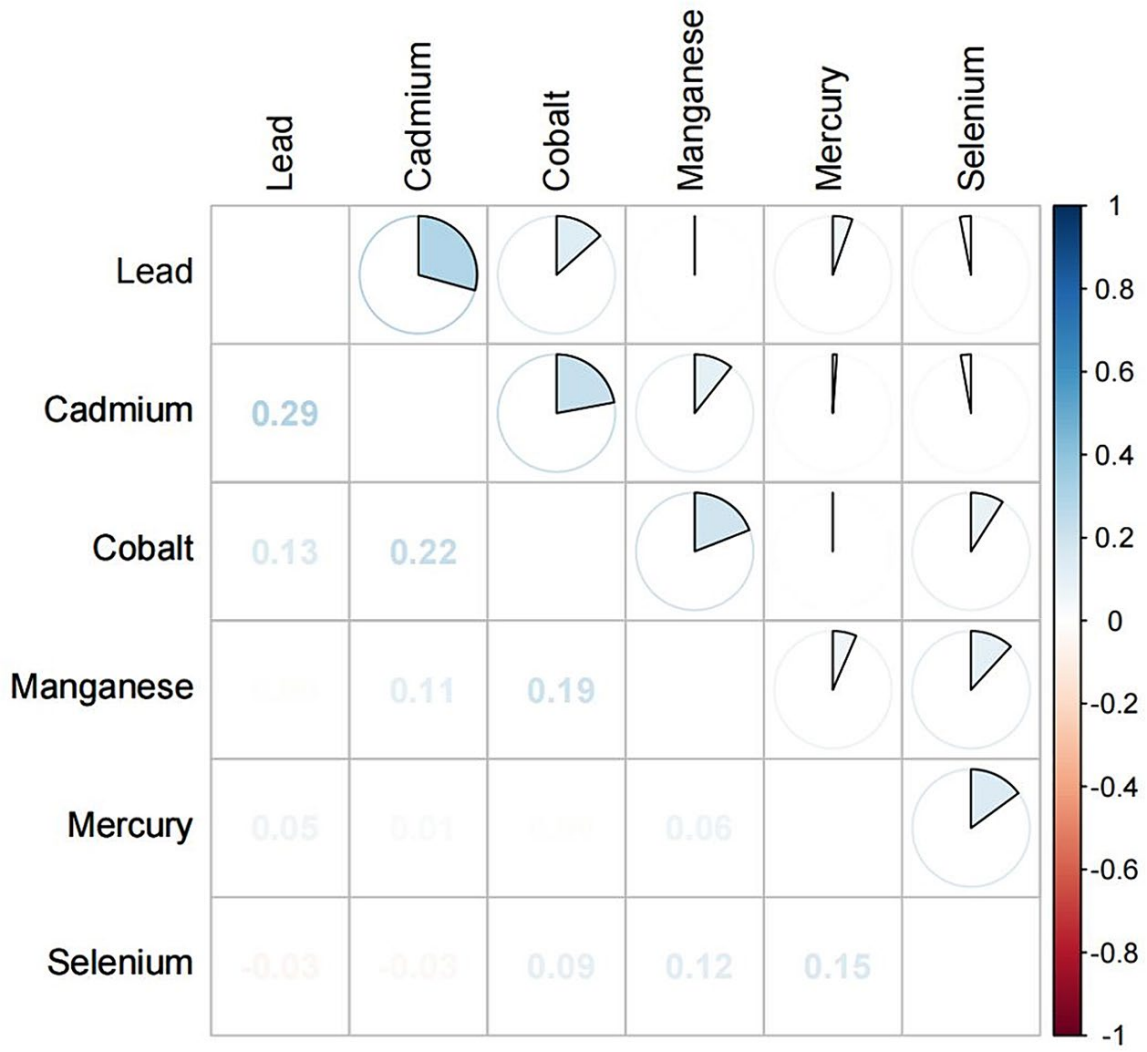
Spearman correlation plot of concentrations of individual metals.

There were significant differences in the serum concentrations of Pb, Cd, Hg, Se, Mn, and Co between the CKD and non-CKD groups. The CKD group had significantly higher concentrations of Pb, Cd, and Co than the non-CKD group. Spearman correlation analysis showed that the correlation coefficients of Cd and Pb, Cd and Co were 0.29 and 0.22, respectively (Figure 2).

### 3.3 Multiple logistic regression analysis

**Figure 3:**
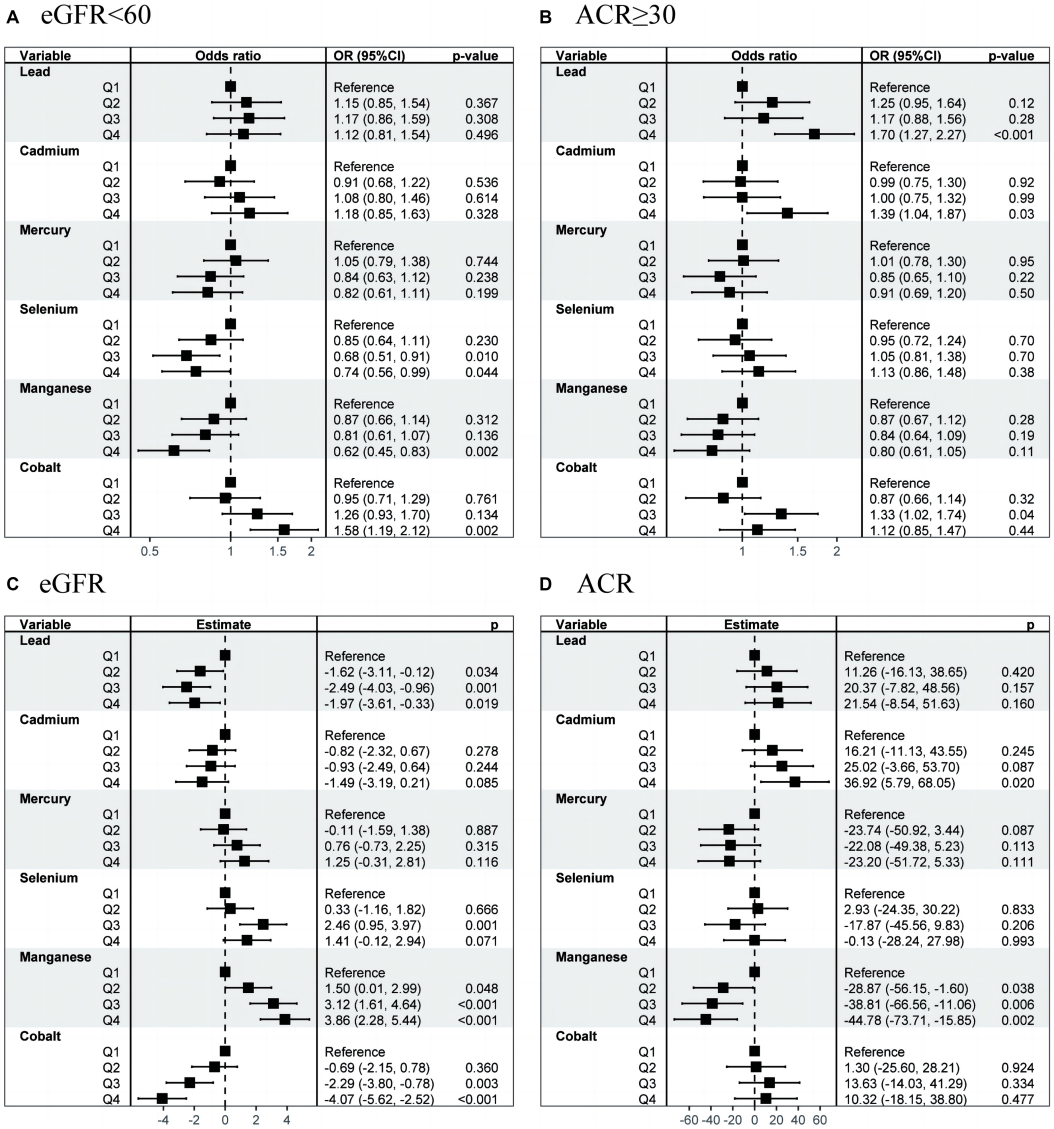
OR (95% CI) in eGFR, CKD, ACR, and proteinuria associated with single metals levels. OR, odds ratio; CI, confidence interval; CKD, chronic kidney disease.

Multiple logistic regression analysis revealed significant correlation between the concentrations of Se (Q3 vs. Q1; OR, 0.68; 95% CI, 0.51–0.91; *p* = 0.010), Mn (Q4 vs. Q1; OR, 0.62; 95% CI, 0.45–0.83; *p* = 0.002), and Co (Q4 vs. Q1; OR, 1.58; 95% CI, 1.19–2.12; *p* = 0.002) and the incidence of CKD (Figure 3A). Meanwhile, high concentrations of Pb (Q4 vs. Q1; OR, 1.70; 95% CI, 1.27–2.27; *p* < 0.001), Cd (Q4 vs. Q1; OR, 1.39; 95% CI, 1.04–1.87; *p* = 0.029), and Co (Q3 vs. Q1; OR, 1.33; 95% CI, 1.02–1.74; *p* = 0.037) were identified as risk factors for proteinuria (Figure 3B). Continuous outcome results also be provided in Figures 3C,D.

### 3.4 WQS analysis

**Figure 4:**
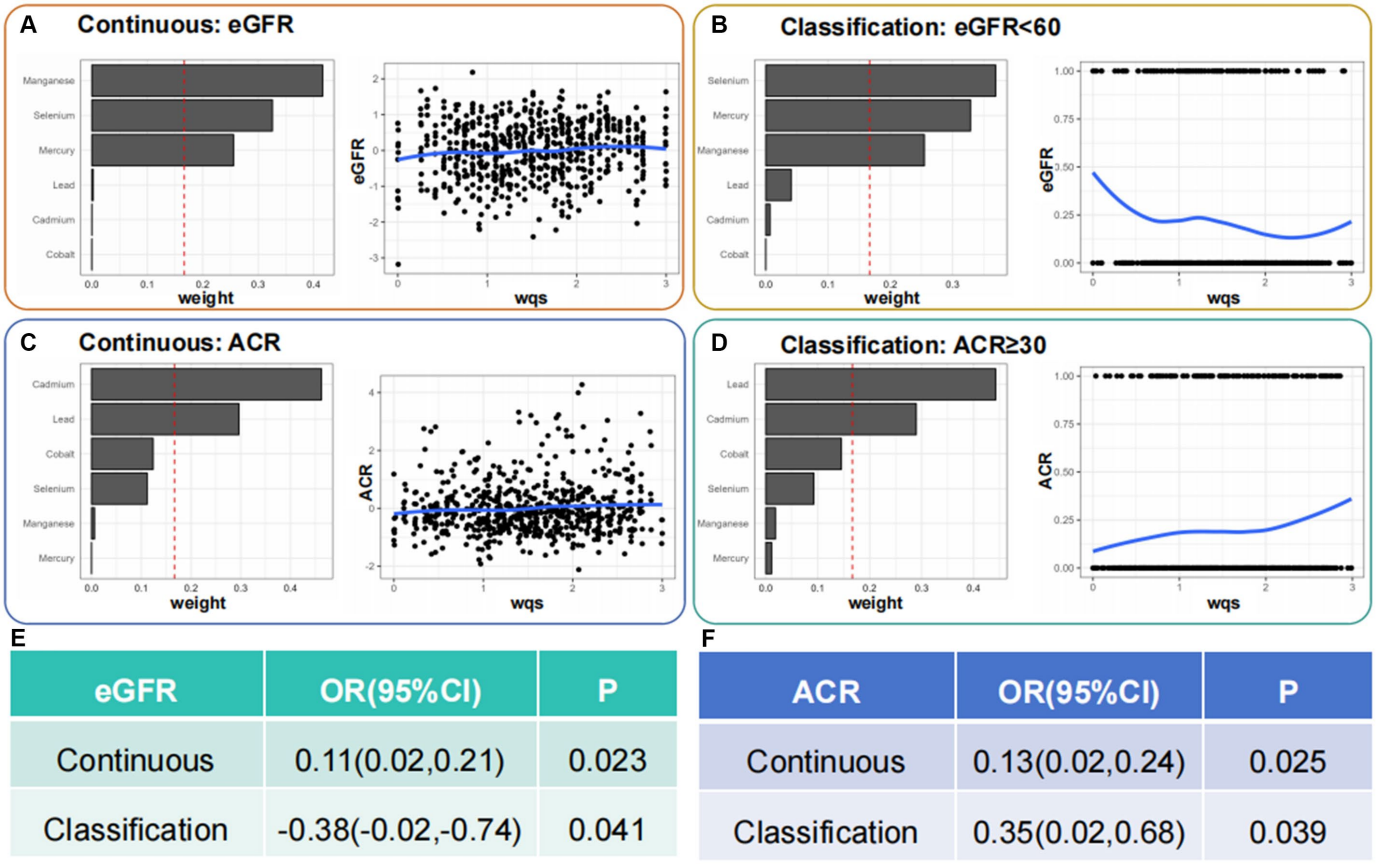
Identification of blood heavy metals in the mixture using the WQS model. WQS, weighted quantile sum.

We preliminarily studied the effects of mixed serum heavy metal exposure on eGFR, ACR continuous variables, and CKD and proteinuria binary variables (Figure 4). Mixed metal exposure was positively correlated with eGFR but negatively correlated with CKD. Se, Hg, and Mn had significant effects on eGFR and CKD. Mixed metal exposure was positively correlated with ACR and proteinuria, and the weights of Pb, Cd, and Co metals were relatively high, consistent with logistic regression results.

### 3.5 BKMR analysis

**Figure 5:**
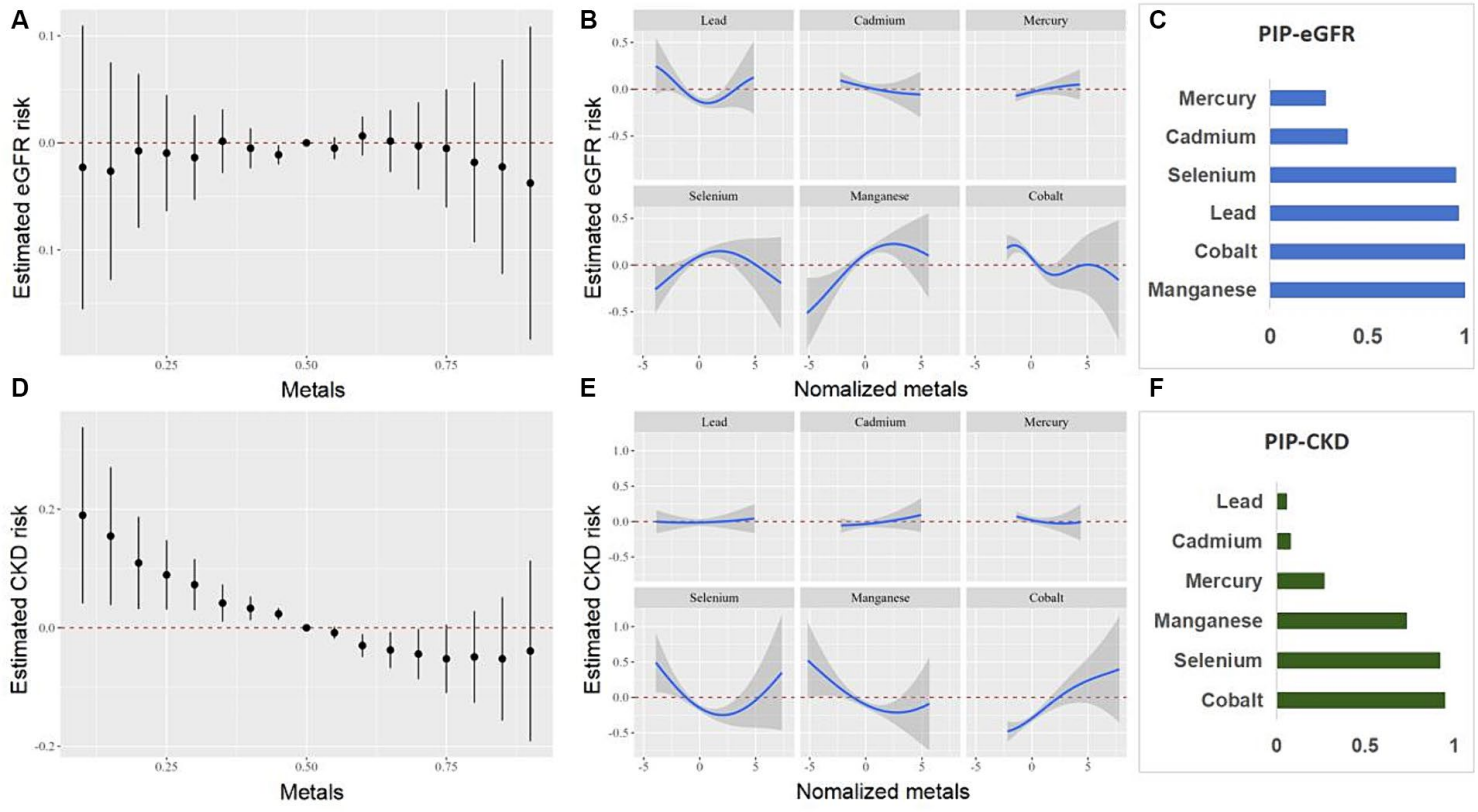
Combined effects of the metals as a mixture on eGFR and CKD in older adult people. eGFR, estimated glomerular filtration rate; CKD, chronic kidney disease.

**Figure 6:**
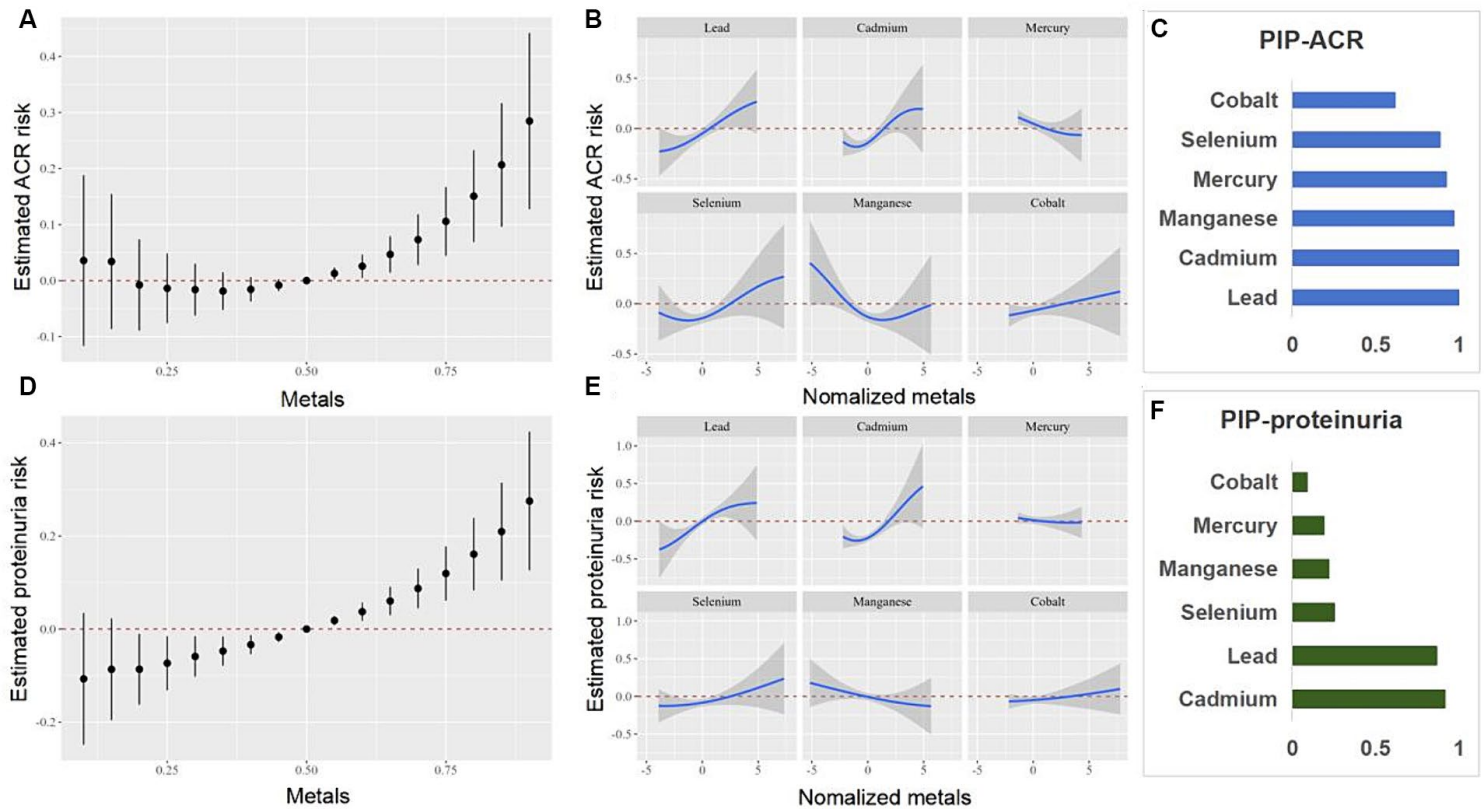
Combined effects of the metals as a mixture on ACR and proteinuria in older adult people. ACR, urinary albumin creatinine ratio.

We further investigated the relationship between the co-exposure to six blood heavy metals and eGFR, CKD, ACR, and proteinuria using the BKMR model (Figures 5, 6). The Figure 5 shows a negative correlation between mixed exposure to six metals and CKD. Co, Se, and Mn had the highest weight proportion in mixed metal exposure. The exposure level of other blood heavy metals was set at the median to evaluate the effect of a single metal exposure reaction function. Co was positively correlated with CKD, Mn was negatively correlated with CKD, and Se was positively U-shaped correlated with CKD. Mixed exposure to six metals was positively correlated with proteinuria in the older adult participants, with Cd, Pb, and Se weighing the highest. Figure 6 shows the correlation between each metal and ACR.

### 3.6 Mediation of UA

**Figure 7:**
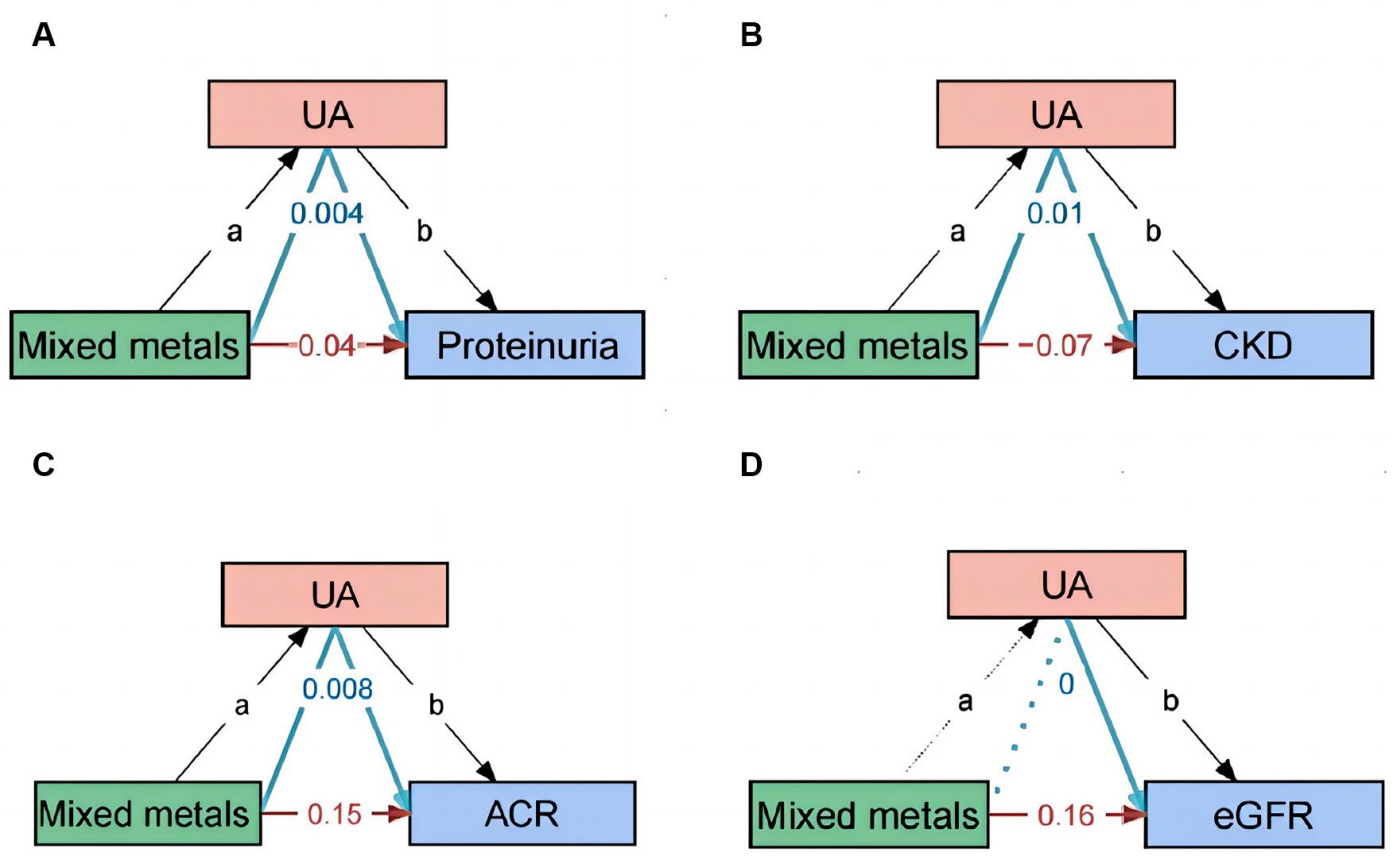
Path diagram of the mediation analysis model. In the mediation analysis, mixed metals are defined as the exposure factor; proteinuria, ACR, eGFR, and CKD are defined as the outcome; and UA is defined as the mediator. CKD, chronic kidney disease; UA, uric acid.

We have performed mediation analyses to assess whether UA mediates the association between metals and the occurrence of proteinuria and CKD. The model and pathway for the mediation analysis are shown in Figure 7. After adjusting for all potential confounders, the results showed approximately 9.7% ([95% CI, 3.5–29.1%]; *p* = 0.003) of the effect of mixed metals exposure on proteinuria was mediated by UA. Approximately −19.7% ([95% CI, −64.2 – −1.9%]; *p* = 0.03) of the effect of mixed metals exposure on CKD was mediated by UA. The mediating effect of UA between mixed metals and eGFR and ACR is also presented in Figure 7.

## 4 Discussion

The study firstly found that mixed exposure to six metals was positively correlated with proteinuria in older adult participants, with UA acting as a mediating factor. Se and Mn were identified as protective factors against CKD, while Co was determined to be a risk factor for the development of CKD. However, this is a cross-sectional study, which cannot conclude the casual relationship between metals and renal function. Longitudinal studies should further evaluate whether the mixed effects of metals and other nephrotoxic substances may be a risk factor for renal injury in older adult people.

Cd, Pb, and Hg are toxic metals generated both naturally and through human activities; these metals can chemically pollute products that enter the human food chain (24, 25). Cd, Pb, and Hg are associated with an increased risk of various age-related chronic diseases, including cardiovascular disease, CKD, and osteoporosis (26–28). Hg, Cd, and Pb ions in the blood bind to thiol-containing biomolecules such as albumin, glutathione, and cysteine to some extent, leading to kidney damage (29). Inflammation is also an important mechanism by which cadmium and other substances cause kidney damage (30, 31). Even low levels of Cd in the kidneys during renal biopsy can induce mild tubular atrophy, and a positive correlation has been reported between renal Hg and renal arteriosclerosis (32, 33). A study from South Korea found no association between Pb, Hg, and Cd levels in the blood of adolescents and eGFR (34). However, a study from China found that both single and mixed exposure to Cd and Pb in adults is associated with renal dysfunction (35). A study from the United States also found that, in adults, excessive exposure to Pb, as well as any level of Cd and total Hg, can have adverse effects on kidney function and health (36). Therefore, the effects of Cd, Pb, and Hg on renal function may vary among age groups. The present study found that Pb, Hg, and Cd were not associated with CKD in the older adult participants, whereas Pb and Cd were associated with proteinuria in the older adult participants. Co—an essential element and a well-known component of cobalamin (vitamin B ₁₂)—has recently been proven to be a mimic of hypoxia and a stimulant for reactive oxygen species production, but it is toxic at high concentrations (37). This study found a close correlation between Co and proteinuria.

There are many studies about the effects and mechanisms of Se and Mn on renal function and proteinuria in participants. Among all human organs, the kidneys and thyroid have the highest Se content (38). Se can enhance antioxidant capacity, primarily by increasing the activity of antioxidant enzymes, such as glutathione peroxidase (39). A placebo-controlled study targeting the older adult population in Sweden demonstrated that low Se status is associated with age-related decline in kidney function. In this study, dietary supplements were administered for 4 years with a Se content of 200 μg. Compared with the functional indicators of the placebo group, there was a significant improvement in renal function with Se and coenzyme Q10 (40). A study on the Se status and CKD of 5,381 middle-aged and older adult people suggests that adequate Se intake may have a positive effect on CKD (41). Our study found that Se has a protective effect on proteinuria and CKD. Mn is crucial for the normal operation of various metabolic enzymes and cofactors. It is also an essential trace element for maintaining normal bodily functions, a cofactor of many enzymes, crucial for substance and energy metabolism, immune function, and blood glucose regulation, and has antioxidant effects (42). Studying plasma levels of Mn, Fe, and Zn can prevent the risk of CKD in older adult people (≥ 90 years) in long-lived areas (10). This study also found that Mn was negatively correlated with the incidence rate of CKD. Another study showed that blood Cu levels are significantly associated with CKD risk, showing a positive dose–response relationship in the Chinese older adult population. Exposure to Mn can antagonize the toxicity of Cu on renal function (43).

The analyses of mixtures, rather than of single metals, may provide a real-world perspective on the relationship between metals and kidney function (44). Previous studies have found that mixed exposure to three nephrotoxic metals (Cd, Pb, and Hg) in the blood is unrelated to eGFR and urinary protein in adolescents (9). Another study reported that exposure to coexisting heavy metal mixtures (Co, Cr, Hg, and Pb) is associated with indicators of poor kidney function in adults (45). Our study found that there is a positive correlation between mixed exposure to six metals and ACR, with higher concentrations leading to greater proteinuria risk, among which Pb and Cr have a larger weight. Moreover, in the univariate analysis, Pb and Cd were identified as risk factors for proteinuria. However, mixed exposure to six metals was positively correlated with CKD, possibly because Pb, Hg, and Cd were not associated with CKD in older adult participants. Although Co was negatively correlated with CKD, Se and Mn accounted for a significant proportion, which could neutralize the kidney damage caused by Co.

Increased serum levels of UA have been associated with the onset and development of CKD, through several molecular pathogenetic mechanisms, such as inflammation and oxidative stress (46). Multiple studies have found a close correlation between heavy metals and high UA levels (19, 47). Our study reveals for the first time that heavy metals can not only directly affect the kidneys, but also regulate renal function by mediating UA levels.

## 5 Conclusion

To our knowledge, the study is the first to investigate the relationship between mixed exposure to six metals (Pb, Cd, Hg, Se, Mn, and Co) and renal function measured in the blood of older adult people, and UA serving as a mediating factor. Our results indicate that both single and mixed metal exposure may affect renal function, although potential reverse causal relationships cannot be ruled out because of the cross-sectional study design. Our results show that the heavy metals in the blood (Pb, Cd, Hg, and Co) are associated with renal function damage, whereas Se and Mn have a protective effect on renal function in older adult people. When exposed to a metal mixture, Se and Mn may counteract the renal damage caused by other metal ions. Longitudinal studies should further evaluate whether the mixed effects of metals and other nephrotoxic substances may be a risk factor for renal injury in older adult people.

## Data availability statement

The datasets presented in this study can be found in online repositories. The names of the repository/repositories and accession number(s) can be found below: NHANES – National Health and Nutrition Examination Survey Homepage (cdc.gov).

## Ethics statement

The studies involving humans were approved by the data comes from publicly available databases (NHANES). The studies were conducted in accordance with the local legislation and institutional requirements. Written informed consent for participation in this study was provided by the participants’ legal guardians/next of kin.

## Author contributions

SP: Investigation, Writing – original draft, Formal analysis. YN: Methodology, Writing – original draft. SD: Writing – review & editing, Investigation. DZ: Methodology, Writing – original draft. QW: Investigation, Writing – original draft. ZD: Conceptualization, Funding acquisition, Writing – review & editing. GC: Conceptualization, Funding acquisition, Supervision, Writing – review & editing. XC: Conceptualization, Funding acquisition, Supervision, Writing – review & editing.
